# The transcription factor RBP-J-mediated signaling is essential for dendritic cells to evoke efficient anti-tumor immune responses in mice

**DOI:** 10.1186/1476-4598-9-90

**Published:** 2010-04-27

**Authors:** Fan Feng, Yao-Chun Wang, Xing-Bin Hu, Xiao-Wei Liu, Gang Ji, Yun-Ru Chen, Lin Wang, Fei He, Guo-Rui Dou, Liang Liang, Hong-Wei Zhang, Hua Han

**Affiliations:** 1 Department of Medical Genetics and Developmental Biology, State Key Laboratory of Cancer Biology, Fourth Military Medical University, 15 West Changle Road, Xi'an, 710032, China; 2 Xijing Hospital of Digestive Diseases, State Key Laboratory of Cancer Biology, Fourth Military Medical University, 15 West Changle Road, Xi'an, 710032, China

## Abstract

**Background:**

Dendritic cells (DCs) are professional antigen presenting cells that initiate specific immune responses against tumor cells. Transcription factor RBP-J-mediated Notch signaling regulates DC genesis, but whether this pathway regulates DC function in anti-tumor immunity remains unclear. In the present work we attempted to identify the role of Notch signaling in DC-mediated anti-tumor immune response.

**Results:**

When DCs were co-inoculated together with tumor cells, while the control DCs repressed tumor growth, the RBP-J deficient DCs had lost tumor repression activity. This was most likely due to that DCs with the conditionally ablated RBP-J were unable to evoke anti-tumor immune responses in the solid tumors. Indeed, tumors containing the RBP-J deficient DCs had fewer infiltrating T-cells, B-cells and NK-cells. Similarly, the draining lymph nodes of the tumors with RBP-J^-/- ^DCs were smaller in size, and contained fewer cells of the T, B and NK lineages, as compared with the controls. At the molecular level, the RBP-J deficient DCs expressed lower MHC II, CD80, CD86, and CCR7, resulting in inefficient DC migration and T-cell activation in vitro and in vivo. T-cells stimulated by the RBP-J deficient DCs did not possess efficient cytotoxicity against tumor cells, in contrast to the control DCs.

**Conclusion:**

The RBP-J-mediated Notch signaling is essential for DC-dependent anti-tumor immune responses. The deficiency of RBP-J impairs the DC-based anti-tumor immunity through affecting series of processes including maturation, migration, antigen presentation and T-cell activation. The Notch signaling pathway might be a target for the establishment of the DC-based anti-tumor immunotherapies.

## Background

Dendritic cells (DCs) are professional antigen presenting cells (APCs) that initiate specific immune responses against pathogens [1] and tumor cells [2]. Immature DCs which locate in the tissues and the peripheral lymphoid organs persistently surveillance the environment and recognize the invading pathogens and cell debris [3], and capture antigens by phagocytosis, micropinocytosis, and endocytosis. After the antigen recognition and uptake, the immature DCs undergo a series of maturation events, including the up-regulation of the major histocompatibility complex (MHC) II and the co-stimulatory molecules, the secretion of cytokines, the outgrowth of dendrites, and the modulation of chemokine receptor expression profile accompanied by the migration into the T-cell areas of the peripheral lymphoid organs [4]. The antigen-loaded mature DCs can activate T-cells through the interaction between MHC II-peptide and T-cell receptor (TCR) complex, and can activate B-cells [5] and NK-cells [6] through specific ligands and cytokines expressed by DCs. Based on the differential expression of cell surface markers, DCs are grouped into two major classes including conventional DCs (cDCs) [7] and plasmacytoid DCs (pDCs) [8]. cDCs are further subdivided into different populations including the lymphoid tissue-resident DCs and the peripheral tissues-located migrating DCs [1,4].

DCs play critical roles in the initiation, programming and regulation of the anti-tumor immunity [9,10]. Nevertheless, as supported by both experimental studies and clinical observations, the immune responses against tumor cells are severely compromised in most, if not all, progressing solid tumors. The tumor infiltrating myeloid-derived suppressor cells (MDSCs), the tumor-associated macrophages (TAMs) [11,12] and the cytokines secreted by MDSCs and TAMs cooperatively create an immunosuppressive environment which leads to the suppression of DC functions and the induction of regulatory T-cells. Gerner et al reported that murine tumors were extensively infiltrated by partially activated tumor-infiltrating DCs (TIDCs) which had inefficient MHC II presentation due to poor intrinsic protein uptake capability, resulting in the inferior initiation of T-cell responses in the draining lymph nodes [13]. These resting, non-activated, immature phenotypes of DCs have also been discovered in cancer patients [14]. Moreover, DCs have been considered as a promising agent to generate effective anti-tumor immune therapies, because DCs can be generated in large numbers, and the cultured immature DCs could be converted into mature DCs through the antigen loading with peptides, recombinant proteins, tumor antigen-encoding mRNA, and whole tumor cell lysates. These DCs can be delivered to the tumor sites or the lymph nodes to activate T-cell responses against tumors [15]. However, although the use of mature DCs as cellular vaccines showed promising anti-tumor effects in many mouse tumor models such as the B16 melanoma [16], the Lewis lung cancer, the D2F2/E2 breast tumor and the EL4/E2 thymoma [17], the application of mature DCs in phase III clinical trails in human cancer patients with prostate cancer [18] or melanoma [15] have largely failed. Therefore, the fully understanding of the molecular mechanisms regulating DC maturation and activation, which is still obscure, is a prerequisite for the DC-based anti-tumor therapies.

The Notch signaling pathway is an evolutionarily conserved pathway that regulates development by participating in cell fate determinations and cell proliferation, differentiation and apoptosis during embryonic and postnatal stages [19]. There are four Notch receptors (Notch1-4) and five ligands (Jagged1, Jagged2, and Delta-like (Dll)1, 3, and 4) in mammals. After the triggering of the Notch receptors by the binding of the Notch ligands, the Notch intracellular domain (NIC) is cleaved by a proteinase complex containing γ-secretase. NIC then translocates into the nucleus, where it interacts with the transcription factor C promoter-binding factor 1/recombination signal-binding protein J/κ (RBP-J) [20,21]. This protein complex will recruit other transcription co-activators, and transactivate the transcription of the target genes such as the Hes family basic helix-loop-helix members [22].

The Notch signaling plays an important role in the DC genesis. Both of the Notch ligands Jagged1 and Dll1 can activate Notch signaling in DCs, but their effect on DC differentiation is different. Dll1-expressing fibroblasts could induce DC differentiation, whereas Jagged1-expressing fibroblasts inhibit DC differentiation and promote the accumulation of immature myeloid cells [23]. Similar results have also been reported by several other groups. For example, Weijzen et al have shown that Jagged1 is able to induce the maturation of the monocyte-derived human DCs [24], while Ohishi et al have also indicated that Dll1 promotes the DC differentiation [25,26]. Cheng et al showed that the differentiation of DCs was significantly impaired in mice expressing Notch1 anti-sense RNA [27] and in a system involving the Notch1 deficient embryonic stem (ES) cells [28]. The deletion of RBP-J in DCs could result in the reduction of the conventional DCs in the spleens of the mice. This decrease is primarily limited to the CD8^- ^DC subset in the marginal zone of the spleens. As CD8^- ^DCs in the marginal zone were found to reside in close contact with Dll1-expressing cells, Dll1 could also be involved in the loss of CD8^- ^DCs [29]. However, Sekine et al showed that the blocking of Dll1 alone had no significant effect on the maintenance of CD8^- ^DCs and the blocking of Dll1, Dll4, Jagged1 and Jagged2 significantly decreased CD8^- ^DCs [30]. pDCs are different from cDCs in the phenotype and the function. Several studies have reported that the differentiation of pDCs could also be affected by Notch signaling. Oliver et al reported that Dll1 could increase the numbers of pDC through promoting the differentiation rather than affecting the proliferation. A γ-secretase inhibitor (GSI) could block this effect. But another group reported the opposite result, showing that Dll1 could block pDC development [31].

We have recently shown that the RBP-J-mediated Notch signaling plays a critical role in the maturation of the LPS-induced DCs [32]. In order to investigate whether the Notch signaling participates in the DC-mediated anti-tumor immunity, we established tumor-bearing mouse models by using several mouse tumor cell lines and RBP-J-deleted DCs [33]. We found that the absence of RBP-J in DCs led to impaired DC-dependent anti-tumor immune responses. We further demonstrated that the RBP-J deficient DCs could not undergo a full activation process upon tumor antigen stimulation, which resulted in an inefficient T-cell activation and tumor progress.

## Results

### Tumor antigen-loaded spleen-derived DCs (SPDCs) repress tumor growth and tune up Notch signaling

**Figure 1:**
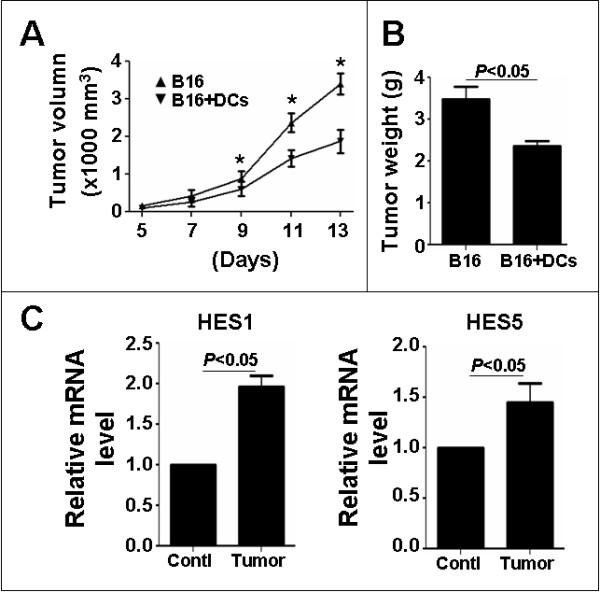
**SPDCs repress the growth of B16 tumors when co-inoculated with tumor cells**. **(A) **The B16 tumor cells (5 × 10^6^) were mixed without or with wild type SPDCs (1 × 10^6^), and were injected subcutaneously into normal mice. The tumor volume was monitored every 2 days from the 5th day after the inoculation, by measuring the tumor length (L) and short (S) with a sliding caliper. Tumor volume = L × S^2 ^× 0.51. **(B) **The tumors were dissected 13 days after the inoculation, and the tumor weight was compared. **(C) **qRT-PCR analysis. DCs in the spleens of wild type and B16 tumor-bearing mice were sorted by anti-CD11c magnetic beads. The Notch downstream genes were analysed by qRT-PCR, with β-actin as an internal control. Bars, means ± SD, * *P *< 0.05 (n = 4).

Mice bearing a B16 malenoma tumor had an enlarged spleen (data not shown), suggesting active immune responses in the spleen. To evaluate the role of SPDCs in anti-tumor immunity, we isolated CD11c^+ ^SPDCs from normal mice, pre-incubated with B16 crude tumor antigens for 12 h, and then mixed SPDCs with B16 cells to subcutaneously inoculate normal mice. We found that compared with tumors without SPDCs, tumors inoculated with SPDCs grew significantly slower regarding tumor size and tumor weight (Fig. 1A and 1B), suggesting that SPDCs had significant tumor-repressing activities.

We and others have recently shown that the RBP-J-mediated Notch signaling is essential for the maturation of peptide or LPS-stimulated DCs [24,32]. Thus, we asked whether Notch signaling pathway was involved in DC-mediated anti-tumor immunity. As an initial step to explore this, the relative mRNA level of HES1 and HES5, two major downstream molecules of Notch signaling, in SPDCs was examined in tumor bearing mice. As shown in Fig. 1C, compared with the control, SPDCs from tumor-bearing mice had higher level of HES1 and HES5, indicating that Notch signaling was activated in SPDCs of tumor-bearing mice.

### RBP-J deficient DCs are unable to repress tumor growth when co-inoculated with tumor cells in mice

To investigate the role of Notch signaling in the DC-dependent anti-tumor immunity, we used the RBP-J conditional deletion mouse model [33]. RBP-J-floxed mice were mated with the Mx-Cre transgenic mice. In the adult RBP-J^+/f^-MxCre (RBP-J^+/-^) and RBP-J^f/f^-MxCre (RBP-J^-/-^) mice, the injection of the interferon (IFN)-α inducer poly(I)-poly(C) induced the deletion of the DNA-binding domain of the floxed RBP-J alleles. The deletion was almost complete in hematopoietic cells including DCs [[32,33], and data not shown]. We then established tumor-bearing mouse models to investigate the effects of RBP-J deletion on the anti-tumor immunity of DCs.

**Figure 2:**
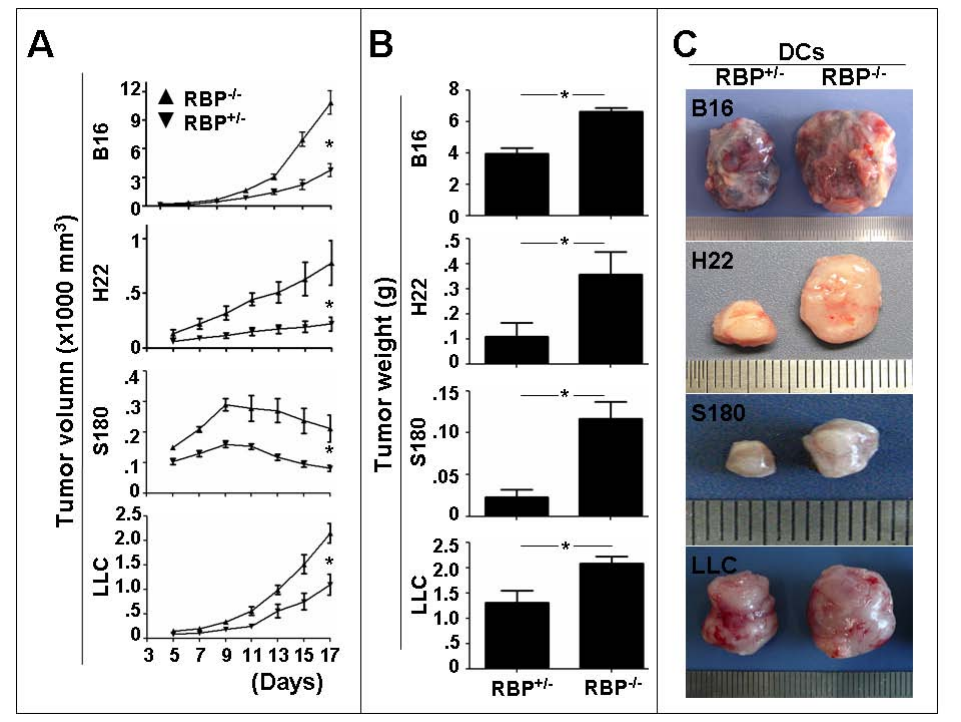
**Tumor growth in mice inoculated with the tumor cells mixed with RBP-J^+/- ^and RBP-J^-/- ^SPDCs**. **(A) **The B16, H22, S180 and LLC tumor cells (5 × 10^6^) were mixed with RBP-J^+/- ^and RBP-J^-/- ^SPDCs (1 × 10^6^), and were injected subcutaneously into normal mice. The tumor volume was monitored every 2 days from the 5th day after the inoculation. **(B) **The tumors were dissected 17 days after the inoculation, and the tumor weight was compared. Bars, means ± SD. *P < 0.05. *n *= 4 in B16; *n *= 3 in H22; *n *= 3 in S180; *n *= 3 in LLC. **(C) **Representative tumors were compared between the two groups.

SPDCs of the RBP-J deleted mice and the control mice were co-injected subcutaneously with four types of the mouse tumor cell lines, including B16, H22, S180, and LLC, into wild type C57BL/6 mice. The growth of the tumors was monitored with two standards, including the tumor volume and the tumor weight. From the 5th day after the inoculation, the tumor volume was followed up to the 17th day. As shown in Fig. 2A, compared with the tumors containing RBP-J^+/- ^DCs, the tumors containing RBP-J^-/- ^DCs grew significantly faster in volume. Even in the S180 sarcoma, of which the tumor size decreases from 9th day after the inoculation, the tumors with the RBP-J deficient DCs were constantly larger than the tumors with the control DCs at all time points examined. Moreover, on day 17 after the inoculation, tumors were dissected and the tumor weight was measured. In all of the four types of tumors, the tumors containing RBP-J^-/- ^DCs were significantly heavier than the tumors containing the control DCs (Fig. 2B). Fig. 2C shows representative tumors dissected on the day 17 after the co-inoculation of tumor cells with different types of DCs. These results indicated that the RBP-J deficient DCs had remarkably reduced tumor-suppressing activities. RBP-J^+/- ^DCs were indistinguishable from the wild type DCs in their anti-tumor activities in this experiment system (data not shown).

SPDCs contain several subtypes of DCs, and RBP-J deletion might influence the differentiation of a specific subtype [29,30]. We examined different subpopulations of SPCDs from RBP-J deficient mice, or irradiated mice after accepting BM cells from RBP-J deficient mice. As shown in additional file 1, while CD8^- ^DCs decreased most remarkably, all subpopulations of SPDCs decreased in the absence of RBP-J. Therefore, attenuated anti-tumor activity of SPDCs could be more likely attributed to impaired DC function, like in LPS or peptide-activated DCs [24,32].

### Decreased infiltration of T, B and NK cells in the tumors containing the RBP-J^-/- ^DCs in mice

**Figure 3:**
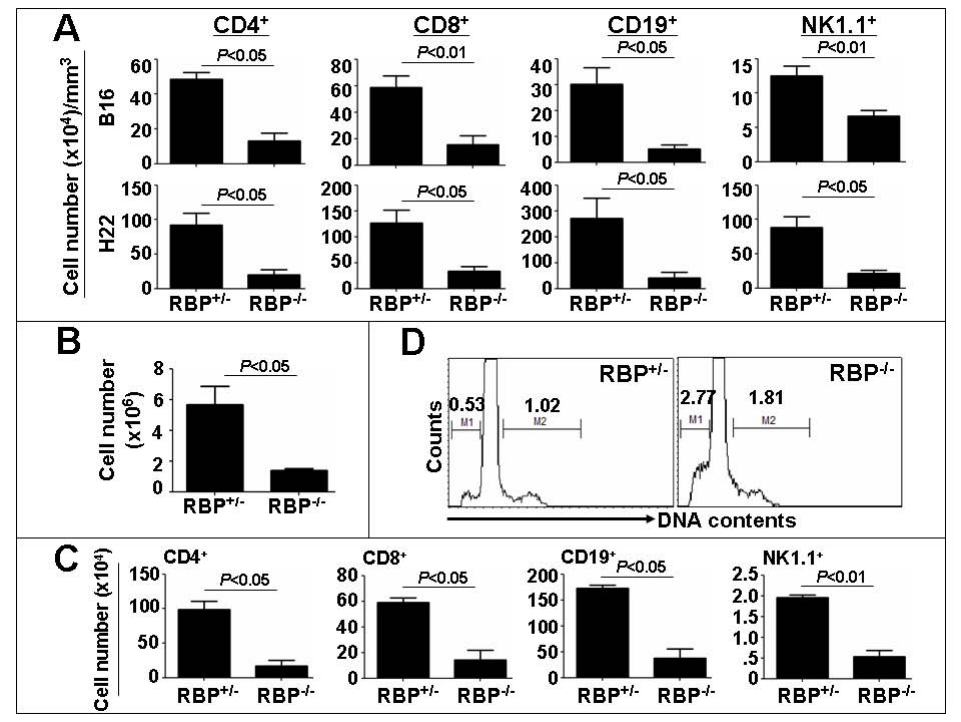
**Infiltration of CD4^+^, CD8^+^, CD19^+ ^and NK1.1^+ ^cells in the tumors and draining lymph nodes of the mice bearing B16 and H22 tumors**. The tumor cells were mixed with RBP-J^+/- ^and RBP-J^-/- ^SPDCs, and were injected subcutaneously into normal mice. **(A) **The tumors were dissected on the 17th day after the inoculation, and the single-cell suspensions were prepared for FACS analyses. The number of CD4^+^, CD8^+^, CD19^+ ^and NK1.1^+ ^cells in 1 g of tumor tissues was calculated based on FACS. **(B) **The draining lymph nodes were dissected, and the total cell number in the lymph nodes was compared between the RBP-J^-/- ^and RBP-J^+/- ^groups. **(C) **FACS was carried out to compared the number of CD4^+^, CD8^+^, CD19^+ ^and NK1.1^+ ^cells in the draining lymph nodes of mice bearing melanoma containing the RBP-J deficient SPDCs or the control SPDCs. Bars, means ± SD. *n *= 4. **(D) **DNA content analysis of the cells isolated from the draining lymph node of the mice bearing melanoma containing the RBP-J deficient DCs or the control DCs. Pictures represent one set of four independent experiments.

DCs function in the initiation of the anti-tumor immune responses through the recruitment and the activation of T-cells [34], B-cells [5] and NK cells [35] into tumors. So, we examined these populations of immune cells in tumors inoculated with the RBP-J deficient and the control DCs in mice. The B16, LLC, H22 and S180 tumor cells were mixed with the RBP-J deficient and the control SPDCs, and were injected subcutaneously into normal mice. Immune cells infiltrating the tumors were examined 17 days after the inoculation by FACS analysis. We found that in the B16 melanoma and the H22 hepatocarcinoma, the infiltration of CD4^+ ^T-cells, CD8^+ ^T-cells, CD19^+ ^B-cells and NK1.1^+ ^NK-cells were significantly lower in tumors containing the RBP-J deficient DCs than in the tumors containing the control SPDCs (Fig. 3A). In the S180 sarcoma, while the infiltrating CD4^+ ^T-cells and CD19^+ ^B-cells were significantly lower in tumors with RBP-J^-/- ^DCs, the decease of CD8^+ ^T-cells and NK-cells appeared not so impressive (see additional file 2). In LLC, only the CD4^+ ^T-cells decreased significantly in the tumors co-inoculated with the RBP-J deficient DCs (see additional file 2). These results suggested that the RBP-J deficient DCs had weaker capacity in the activation and/or recruitment of at least some populations of immune cells into solid tumors.

### Inefficient activation of immune cells in the draining lymph nodes of mice bearing tumors containing RBP-J deficient DCs

Immune responses taking place in the draining lymph nodes are critical for the establishment of adaptive immunities against tumors, because after the uptake of the tumor antigens, DCs migrate into the draining lymph nodes where DCs present the specific tumor antigens to the naïve T-cells to initiate the adaptive immune responses. We therefore examined the immune responses in the draining lymph nodes of B16 malenoma tumors. The draining lymph nodes of the B16 melanoma containing RBP-J^-/- ^DCs were significantly smaller in size than those of the tumors containing the RBP-J^+/- ^DCs (data not shown). Consistently, the total cell number of the draining lymph nodes of the B16 melanoma containing RBP-J^-/- ^DCs was significantly smaller than that of the tumors containing RBP-J^+/- ^DCs (Fig. 3B). We further analyzed the number of different populations of cells in the draining lymph nodes of melanoma tumors by flow cytometry. The results showed that while the ratio between different cell populations was not significantly skewed (data not shown), the absolute cell numbers of the CD4^+ ^T-cells, CD8^+ ^T-cells, CD19^+ ^B-cells and NK1.1^+ ^NK-cells were significantly lower in the draining lymph nodes of the B16 melanoma containing RBP-J^-/- ^DCs (Fig. 3C). Moreover, we performed the DNA content analysis of cells in the draining lymph nodes by FACS. We found that in the draining lymph nodes of the B16 melanoma containing RBP-J^-/- ^DCs, cells with hyper-diploid DNA amount increased about 77.5%, but cells with hypo-diploid amount of DNA increased 422.6%, compared with the controls (Fig. 3D). These data suggested that the cell apoptosis in the draining lymph nodes of the RBP-J^-/- ^DC-containing B16 melanoma was much higher than that of the controls.

### Decreased CCR7 expression and migration ability of RBP-J deficient DCs

**Figure 4:**
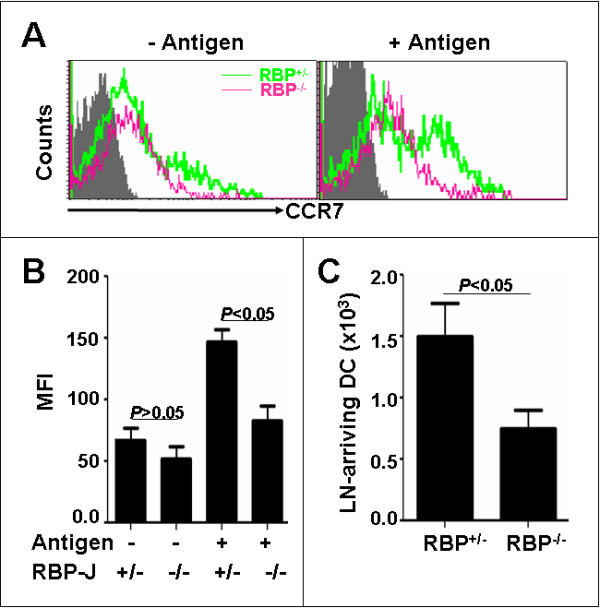
**Cell migration assay**. (**A, B) **SPDCs with different genotypes were co-cultured with the B16 tumor antigens, and the expression of CCR7 was analyzed by flow cytometry. The mean fluorescence intensity (MFI) of CCR7 expression was compared between groups. Bars, means ± SD. *P < 0.05. *n *= 3. **(C) **SPDCs with different genotypes were stained with CFSE, and were incubated with the B16 tumor antigens. SPDCs (1 × 10^6^) were then injected subcutaneously at the left lower abdomen, and the draining lymph nodes were dissected 12 h later. CFSE positive DCs were detected by FACS, and the number of CFSE positive DCs in the draining lymph nodes was calculated based on FACS. Bars, means ± SD. *n *= 3.

We have recently shown that the disruption of RBP-J in BM-derived DCs down-regulated the expression of the chemokine receptor CXCR4 and the chemotactic migration of DCs [32]. We then examined the expression of chemokine receptors on the tumor antigen-loaded DCs derived from the spleens of the RBP-J deficient and the control mice. Crude tumor antigens were prepared using the B16 melanoma cells, and were loaded onto DCs isolated from the spleens of the RBP-J deficient and the control mice. The ability of antigen uptake by DCs appeared comparable between the RBP-J deficient DCs and the control DCs (data not shown). Interestingly, we found that the CXCR4 expression was not changed significantly when compared between RBP-J^+/- ^and RBP-J^-/- ^DCs (data not shown), but the expression of CCR7, a critical chemokine receptor involved in the migration of the antigen-activated DCs, was significantly down-regulated (Fig. 4A and 4B). Then, we performed the in vivo migration assay to analyze the migration ability of RBP-J^-/- ^DCs. DCs isolated from the spleens of the RBP-J^+/- ^and RBP-J^-/- ^mice were labeled with CFSE, and were loaded with the B16 tumor antigens. DCs were injected subcutaneously at the left lower abdomen of the wild type C57BL/6 mice. DCs arriving at the draining lymph nodes were examined by FACS 12 h later. As shown in Fig. 4C, significantly less RBP-J^-/- ^DCs arrived at the draining lymph nodes, compared with RBP-J^+/- ^DCs. These data suggested that the disruption of RBP-J in the SPDCs damaged their migration capacity after the tumor antigen uptake.

### RBP-J deficient DCs show attenuated expression of molecules related to antigen presentation capacities after the loading of tumor antigens

**Figure 5:**
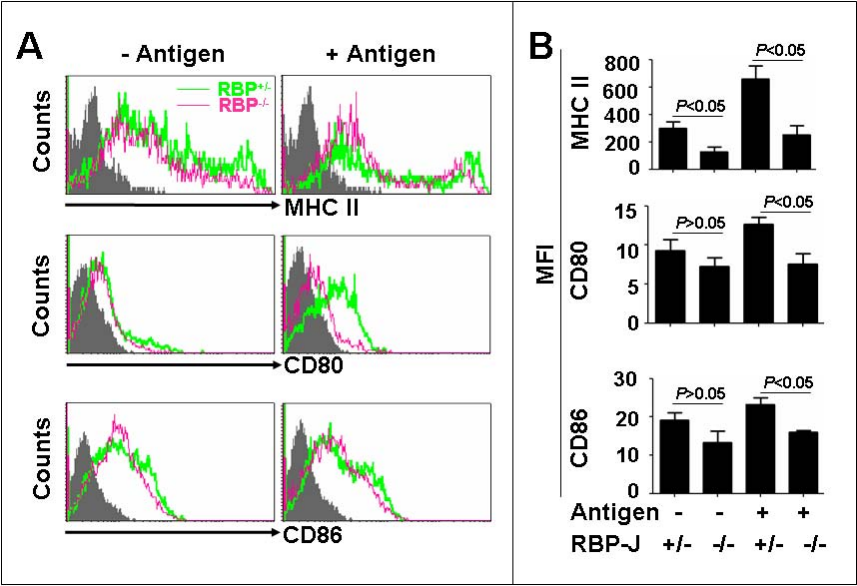
**FACS analysis of MHC II, CD80 and CD86 expressions**. SPDCs with different genotypes were co-cultured with the B16 tumor antigens, and the expression of MHC class II, CD80 and CD86 was analyzed by flow cytometry (A). MFI of MHC class II, CD80 and CD86 expression was compared between groups (B). Bars, means ± SD. *n *= 3.

The antigen presentation by DCs is persuaded by different groups of molecules including MHC I and II and the co-stimulatory molecules. We next examined the expression of these molecules by the antigen-stimulated or the antigen-unstimulated RBP-J^+/- ^and RBP-J^-/- ^DCs. The average expression of MHC II was lower on the RBP-J deficient DCs than the control DCs, regardless with or without antigen stimulation (Fig. 5A and 5B, upper panels). The expression of CD80 and CD86 co-stimulatory molecules were also decreased in the RBP-J^-/- ^DCs compared with the RBP-J^+/- ^DCs after the tumor antigens stimulation (Fig. 5A and 5B, middle and lower panels). These results suggested that RBP-J^-/- ^DCs were not fully activated after the tumor antigen stimulation.

### RBP-J deficient DCs show multiple defects in the activation of anti-tumor T-cells

**Figure 6:**
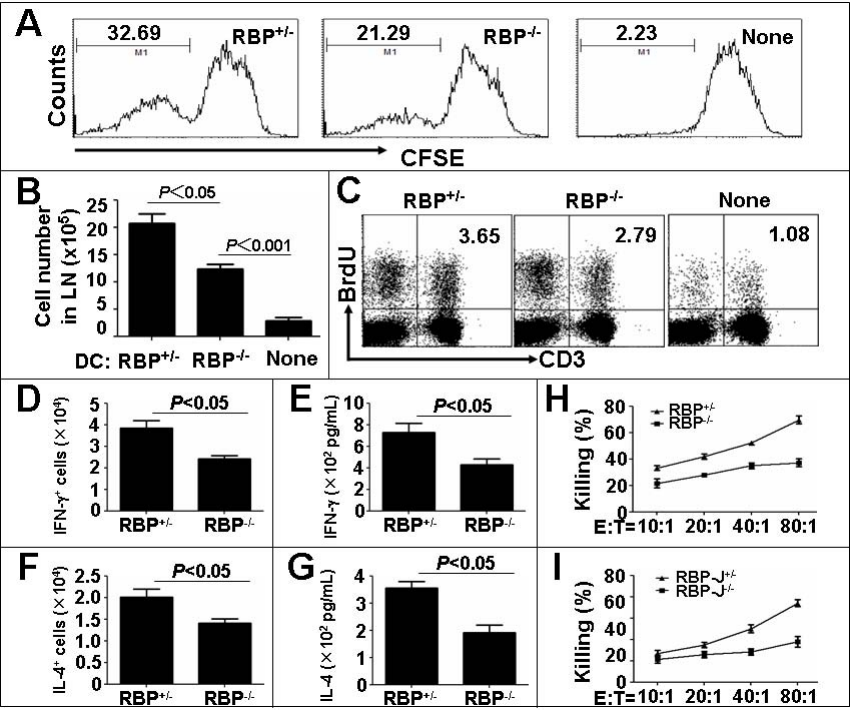
**Decreased proliferation and cytotoxicity of T-cells stimulated with RBP-J deficient SPDCs**. **(A) **FACS analysis. The CFSE-labeled T-cells (1 × 10^6^) were co-cultured with SPDCs (2 × 10^5^) pre-incubated with B16 tumor antigens for 5 days, and the T-cell proliferation was analyzed. **(B) **SPDCs (1 × 10^6^) pre-incubated with the B16 tumor antigens were injected subcutaneously at the lower left abdomen of normal mice. The draining lymph nodes were dissected 7 days later, and the total cell number in the lymph nodes was compared between the RBP-J^-/- ^and RBP-J^+/- ^groups. **(C) **BrdU incorporation assay. SPDCs (1 × 10^6^) pre-incubated with the B16 tumor antigens were injected subcutaneously at the left lower abdomen of normal mice. BrdU was injected intraperitoneally twice per day from the 5th day for 3 days. BrdU^+^CD3^+ ^cells in the draining lymph nodes were detected by flow cytometry 7 days later. **(D-G) **Cytokine production. Naïve T-cells were co-cultured with SPDCs pre-loaded with B16 tumor antigens for 72 h. Cytoplasmic staining was performed to analyze IFN-γ(D) and IL-4 (F) production of T-cells by flow cytometry. ELISA was carried out to detect IFN-γ (E) and IL-4 (G) secretion in the supernatants. **(H) **CTL assay. Normal T-cells were co-cultured with SPDCs pre-loaded with B16 tumor antigens for 3 days. Live B16 melanoma cells were then co-cultured with the activated T-cells at different ratios of 1:10, 1:20, 1:40, 1:80 for 36 h. LDH in the supernatants was detected by using an LDH kit. **(I) **CTL assay. DCs (1 × 10^6^) pre-loaded B16 tumor antigens were injected subcutaneously at the left lower abdomen of normal mice. Seven days later, the draining lymph nodes were dissected and single-cell suspensions were obtained. T-cells were magnetically sorted and were co-cultured with live B16 melanoma cells at different ratios of 10:1, 20:1, 40:1, 80:1 for 36 h. LDH activity in supernatants was detected. Bars, means ± SD. *n *= 3.

We next characterized the T-cell activation ability of the RBP-J deficient and the control SPDCs loaded with B16 tumor antigens. Naïve CD3^+ ^T-cells were obtained from wild type C57BL/6 mice by the negative magnetic selection and were labeled with the CFSE dye. The T-cells were co-cultured with the RBP-J deficient or the control DCs, which had been loaded with the crude B16 tumor antigens. Five days after the starting of the co-culture, the proliferation of T-cells was analyzed for the CFSE dilution by flow cytometry. As shown in Fig. 6A, both of the RBP-J^+/- ^and RBP-J^-/- ^DCs could stimulate T-cell proliferation, but the RBP-J^-/- ^DCs showed a significantly decreased ability in the stimulation of the T-cell proliferation as compared to the control DCs.

To explore this phenomenon in vivo, we performed the BrdU incorporation assay. The B16 tumor antigen-loaded SPDCs were injected subcutaneously at the left lower abdomen of the mice, and BrdU was injected intra-peritoneally on the fifth day after the injection of SPDCs. Seven days later, the mice were sacrificed. The total cell number of the draining lymph nodes of the mice accepting tumor antigen-loaded RBP-J deficient SPDCs was smaller than that of the control (Fig. 6B). The cells of the draining lymph nodes were further analyzed for the DNA content and the BrdU incorporation by the staining with FITC-anti-BrdU and APC-anti-CD3. The results showed that compared with the controls, fewer cells from the draining lymph nodes of the mice accepting RBP-J deficient SPDCs were in the S or G2+M phase of the cell cycle (data not shown). Consistently, the BudU incorporation analysis of the CD3^+ ^T-cells confirmed that the T-cells in the draining lymph nodes of the mice accepting the tumor antigen-loaded RBP-J deficient SPDCs showed less DNA synthesis (Fig. 6C). These results suggested that tumor antigen-loaded RBP-J deficient SPDCs had less activity in the stimulation of T-cells in vivo.

We then examined the secretion of cytokines by the T-cells stimulated with the RBP-J^+/- ^and RBP-J^-/- ^SPDCs loaded with the tumor antigens. Cytoplasmic staining and ELISA showed that the RBP-J deficient SPDCs stimulated less IFN-γ secretion by T-cells, as compared with the control SPDCs (Fig. 6D and 6E). IL-4 secretion by the T-cells stimulated with RBP-J^-/- ^DCs was also lower than T-cells stimulated with the control SPDCs (Fig. 6F and 6G), suggesting that the reduced IFN-γ production by the RBP-J^-/- ^SPDC-stimulated T-cells was not due to a bias of Th1 versus Th2 polarization.

To access the ability of the RBP-J deficient and the control SPDCs to activate cytotoxic T-cells, naïve T-cells were co-cultured with the B16 tumor antigen-loaded SPDCs. After co-cultured for 3 days, the activated T-cells were mixed with B16 tumor cells, and specific cytotoxicity was assessed by the LDH assay. As shown in Fig. 6H, the specific tumor cell lysis ability of the T-cells activated by RBP-J^-/- ^SPDCs was significantly lower than that of the T-cells activated by RBP-J^+/- ^SPDCs. We also performed in vivo CTL assay to investigate the function of the T-cells activated by different SPDCs. Normal mice were vaccinated with the B16 tumor antigen-loaded RBP-J^-/- ^and RBP-J^+/- ^SPDCs. Seven days after the vaccination, T-cells were collected from the draining lymph nodes by negative magnetic selection, and were co-cultured with the B16 tumor cells. We found that the specific tumor cell lysis ability of the T-cells from the RBP-J^-/- ^SPDC-vaccinated mice were significantly lower than that of the T-cells from the control SPDC-vaccinated mice (Fig. 6I), and that the RBP-J^-/- ^SPDC-vaccinated mice possessed smaller draining lymph nodes and spleens as compared with the controls (data not shown). Therefore, we concluded that the RBP-J deficient SPDCs had less capacity to activate T-cells, concerning the cell proliferation, cytokine production, and cytotoxicity.

## Discussion and Conclusion

As the strongest professional antigen presenting cells, DCs play critical roles in the activation of the anti-tumor immunity. Much attention has been paid to the utilization of DC vaccines to initiate efficient tumor specific effector T-cells to inhibit tumor growth. Indeed, in 1990s, many investigators have attempted to use tumor antigen-loaded DCs in the clinical trials of established tumors after the initial success with the preventive and therapeutic DC vaccination in mouse models [36]. Although the use of mature DCs as cellular vaccines had provided encouraging and exciting anti-tumor effects in many mouse tumor models [16,17], the applications of mature DCs in the phase III clinical trails in human cancer patients with prostate cancer [18] or melanoma [15] have largely failed. Obviously, the understanding of the molecular mechanisms regulating DC maturation is essential for the DC-based anti-tumor therapies.

Notch signaling pathway might influence tumor growth in multiple ways [37]. In this study, we showed that the disruption of the transcription factor RBP-J, which is critical in the mediation of the canonical Notch signaling pathway, attenuated the DC-dependent anti-tumor immunities. By using co-inoculation of four types of tumor cells with DCs in mice, which mimic the injection of activated DCs directly into tumor tissues, we found that tumors co-inoculated with the RBP-J deficient DCs were significantly bigger than tumors containing the control DCs. The inefficient infiltration of the immune cells including T-cells, B-cells and NK cells into the tumor tissues containing RBP-J^-/- ^DCs further suggested that DCs with Notch signaling deficiency could not prime adaptive immune responses to tumor cells. Although previous studies have shown that Notch signaling affects the development, differentiation and function of DCs, the results reported here directly showed that the Notch signaling pathway is important for DCs to evoke efficient anti-tumor immune responses in mice.

The phenotypes of the tumor antigen-loaded RBP-J deficient DCs are reminiscent of immature DCs. DCs are derived from hematopoietic stem cells accommodated in BM. Immature DCs are generated through a series of complicated differentiation steps in BM, and are exported into the blood stream and thereafter enter into the secondary lymphoid organs and the peripheral tissues. Normally, upon the uptaking and processing of tumor antigens in the peripheral tissues, immature DCs mature into fully activated DCs, by the gain of migration capacity, the outgrowth of dendrites, and the up-regulated expression of the MHC molecules and the co-stimulatory molecules. Mature DCs subsequently migrate into the draining lymph nodes where they present the processed antigens to resting T lymphocytes in the shape of peptide-MHC complex, to generate immune responses against pathogens and tumors. In vitro and in vivo studies have shown that DCs loaded with tumor antigens could be activated to initiate anti-tumor immune responses. In our study reported here, DCs isolated from the spleen of mouse were activated by the incubation with the crude tumor antigens, and these DCs appeared to have the ability to activate anti-tumor immunities. While the RBP-J^+/- ^DCs showed the properties of mature DCs upon antigen loading, RBP-J^-/- ^DCs appeared immature in several aspects. First, the migration of the tumor antigen-loaded RBP-J^-/- ^DCs was impaired. We analyzed the expression of chemokine receptors including CCR7 and CXCR4, and found that although the CXCR4 level was almost the same in both RBP-J^-/- ^and RBP-J^+/- ^DCs after tumor antigen loading, the expression of CCR7 was significantly lower in RBP-J^-/- ^DCs. CCR7 has been shown to play important roles in the migration of mature DCs. It is likely that the lowered expression of CCR7 mediated the reduced migration of RBP-J deficient DCs from the tumor tissues to the draining lymph nodes, but more detailed molecular experiments are needed to clarify the mechanism controlling the chemokine receptor expression in DCs. Second, the expression of MHC II was weaker in the RBP-J deficient DCs as compared with the control DCs. The up-regulation of the MHC II expression is a critical marker of DC maturation. DCs in our experimental system appeared heterogenous concerning the MHC II expression. In the tumor antigen-unloaded and loaded RBP-J^-/- ^DCs, the level of MHC II was lower than the control DCs. Third, the level of co-stimulatory molecules, CD80 and CD86, was significantly lower on the tumor antigen-loaded RBP-J^-/- ^DCs than that on the control DCs. This is especially true for the expression of CD80. Lowered expression of the co-stimulatory molecules also supports that RBP-J deficient DCs are immature after tumor antigen loading. Last but not least, RBP-J deficient DCs showed lowered capacity in the stimulation of T-cells. Our data showed that both in vitro and in vivo, the tumor antigen-loaded DCs had attenuated capacity of stimulating the T-cell proliferation, cytokine production and cytotoxicity. Interestingly, in the draining lymph nodes of the mice bearing the B16 melanoma containing RBP-J^-/- ^DCs, higher proportion of apoptotic cells was observed, as compared with the controls. This suggested that RBP-J^-/- ^DCs might have immuno-repressive activity, but further studies are needed to access cell proliferation and apoptosis in different populations of cells. The molecular mechanisms, such as the components of crude tumor antigen preparations and the involvement of TLRs, are now under investigation, although the expression of PD-L1 and PD-L2 on DCs was not changed (data not shown). Because no difference was observed in the rate of tumor antigen phagocytosis between the RBP-J deficient and the control DCs (data not shown), the immature phenotypes of the RBP-J deficient DCs could be attributed to some intrinsic defects in the maturation of the tumor antigen-loaded DCs. However, we could not exclude a possibility that RBP-J deficient DCs represent another population of DCs, such as regulatory DCs. Further studies with DC-specific RBP-J deletion mouse models [29] and genome-wide transcription profiling are needed to clarify this point.

B-cells and NK cells were also activated by DCs. Qi et al reported that lymph node B-cells could be activated by antigen-bearing DCs by two-photon intravital imaging [38]. Kijima et al reported that NK-cells could be activated by DCs and that Jagged2-Notch interaction is very critical in this process [6]. Our results also showed that the proportion and absolute number of B cells and NK cells were significantly lower in RBP-J^-/- ^DCs, indicating that Notch signaling is also important for DCs to activate B-cells and NK cells in the anti-tumor immunity.

Taken together, the Notch signaling pathway could affect the differentiation and the maturation of DCs, and the deficiency of Notch signaling pathway could impair DC-based anti-tumor immunity. Our results suggest the important role of Notch signaling pathway in the DC-based anti-tumor immunity. Therefore, the Notch signaling might be a potential target to interfere the DC-based anti-tumor immunotherapies. However, detailed studies about the mechanisms of the regulation of DCs by Notch signaling should be unveiled before the related therapies could be achieved.

## Methods

### Mice

Mice were maintained in the specific pathogen-free (SPF) conditions on the C57BL/6 background. The RBP-J-floxed (RBP-J^f^) mice were as described [33] and were crossed with the Mx-Cre transgenic mice to get the RBP-J^f/f^-MxCre and RBP-J^+/f^-MxCre (as controls) mice (hence, referred as RBP-J^-/- ^and RBP-J^+/-^, respectively). The mice were genotyped by the polymerase chain reaction (PCR) [33]. Four-week-old mice were injected intraperitoneally with 300 μg/100 μl poly(I)-poly(C) (Sigma, St. Louis, MO) for four times at 2-day intervals and were then injected with the same dosage of poly(I)-poly(C) for another eight times at 1-week intervals (twelve injections in total). All animal experiments were approved by the Animal Experiment Administration Commission of Fourth Military Medical University.

### Cell lines and cell culture

Lewis lung carcinoma (LLC), mouse S180 sarcoma (S180), mouse H22 hepatocarcinoma (H22), and mouse melanoma (B16) cell lines were gifts from CH Shi. All of the tumor cell lines were maintained in RPMI 1640 medium supplemented with 10% fetal bovine serum, 2 mM L-glutamine, 50 IU/ml penicillin, and 50 μg/ml streptomycin sulfate.

DCs were separated by anti-CD11c magnetic beads (Miltenyi Biotec GmbH, Germany) from mouse spleen cell suspensions according to the instructions of the manufacturer, and were cultured in RPMI 1640 medium as described previously [32].

### Preparation of crude tumor antigens [39]

The cultured LLC, S180, H22 and B16 cells were harvested by using a cell scraper, washed in phosphate-buffered saline (PBS), and were resuspended in PBS at approximately 5 × 10^7^/mL. The cells were disrupted by freeze and thaw for 5 times, followed by centrifugation in a benchtop microcentrifuge for 10 min at 10,000 rpm. The supernatants were transferred into sterilized Eppendorf tubes, and were stored at -80°C for later use. In some experiments, B16 cells were labeled with the Dio Cell-Labeling Solution (Invitrogen, Carlsbad, CA) according to the manufacturer's protocol, and were then used for the preparation of the Dio-labeled B16 tumor antigens.

### Tumor-bearing mouse models

Tumor cells (5 × 10^6^) were injected subcutaneously into the normal mice. Five days after the initial inoculation, tumor growth was monitored every 2 days by measuring the tumor length (L) and short (S) with a sliding caliper. The tumor size was calculated as L × S^2 ^× 0.51. Seventeen days after the initial inoculation, the tumors were excised and the tumor weight was measured. The tumor tissues and the tumor draining lymph nodes were minced and were filtrated through a nylon filter for flow cytometry analysis.

### Flow cytometry

Single cell suspensions were resuspended with PBS containing 2% fetal calf serum and 0.05% NaN_3 _and were counted. Cells (3-5 × 10^5^) were stained with antibodies at 4°C for 30 min before being analyzed using a FACSCalibur (BD Immunocytometry Systems, San Jose, CA). Dead cells were excluded by the propidium iodide (PI) gating. Data were analyzed using the CellQuest software. PE-anti-CD4 (RM4-5), APC-anti-CD8 (53-6.7), APC-anti-CD19 (1D3), biotinylated anti-NK1.1 (PK136), APC-anti-CD3 (145-2C11), biotinylated anti-IA^b ^(KH174), and FITC-anti-BrdU were purchased from BD PharMingen (San Diego, CA). FITC-anti-CD11c (N418), biotinylated anti-CD11c, FITC-anti-CD80 (16-110A1), PE-anti-CD86 (GL-1), and Streptavidin-APC were products of BioLegend (San Diego, CA), and Streptavidin-PE and biotinylated anti-CCR7 (4B12) were from eBioscience (San Diego, CA).

### Migration assay

DCs (1 × 10^6^) were separated by anti-CD11c magnetic beads from the mouse spleen cell suspensions, and were labeled with CFSE. DCs were co-cultured with the B16 tumor antigens at a ratio of 1:2 for 12 h at 37°C, and were then collected and resuspended in 200 μl PBS, and were injected subcutaneously at the left lower abdomen of mice. The mice were sacrificed 12 h later, and the draining lymph nodes were collected for FACS analysis.

### T-cell proliferation assay

DCs (2 × 10^5^) were seeded in 96-well plates and were co-cultured with B16 tumor antigen for 12 h, followed by medium exchanging. Congeneic T-cells (1 × 10^6^) were isolated by negative selection using magnetic beads, labeled with carboxyfluorescein diacetate succinimidyl ester (CFSE), and were co-cultured with B16 tumor antigen-loaded DCs. Five days later, T-cell proliferation was detected by FACS.

For the in vivo assay, DCs (1 × 10^6^) were sorted by using the anti-CD11c magnetic beads, and were loaded with the B16 tumor antigens for 12 h at 37°C. The tumor antigen-loaded DCs were injected subcutaneously into normal congenic mice at the left lower abdomen. On the 5th day, the mice were injected intraperitoneally with 1 mg/100 μl BrdU twice per day for three days, with PBS as a control. The mice were sacrificed on the 7th day and the draining lymph nodes were dissected. Single cell suspensions were prepared and were stained for CD3 and BrdU (anti-BrdU-FITC) according to the manual of the BrdU Flow kit provided by the manufacturer (BD Biosciences-Pharmingen).

### Enzyme-linked immunosorbent assay (ELISA)

Culture supernatants were collected and were used to detect the level of cytokines using different assay kits (Bioresun, China). OD_450 _was recorded using a spectrophotometer, and was compared between groups.

### CTL assay

DCs were loaded with the B16 tumor antigens for 12 h, and were then co-cultured with negatively selected T-cells (1 × 10^6^) in 96-well plates for 72 h. The culture supernatants were discarded, and the B16 tumor cells were added into the wells with different ratios of 1:10, 1:20, 1:40, 1:80 to T-cells. After co-culturing for 36 h in the serum free RPMI 1640, the supernatants were collected for the examination of cell death by using a colorimetric lactate dehydrogenase (LDH) assay kit (Cayman Chemical Company, Ann Arbor, MI) according to the manufacturer's manuals.

For the in vivo assay, the B16 tumor antigen-loaded DCs (1 × 10^6^) were injected subcutaneously into normal C57BL/6 mice at the left lower abdomen. On the 7th day after the injection, the mice were sacrificed and the draining lymph nodes were recovered. Single cell suspensions were prepared, and T-cells were negatively selected by using magnetic beads. The T-cells (1 × 10^6^) were then co-cultured with the target B16 tumor cells at different ratios of 10:1, 20:1, 40:1, 80:1 in 96-well plates. The cells were co-cultured in the serum free RPMI 1640 for 36 h, and the supernatants were collected for the detection of the cell death by using the LDH assay kit (Cayman Chemical Company).

### Analysis of DNA content

Cells were collected from the draining lymph nodes of the tumor-bearing mice or the mice inoculated with the tumor antigen-loaded DCs. The cells (1 × 10^6^) were fixed with 70% ethanol for 20 min at room temperature, and were then washed once with PBS. After being resuspened in 500 μl of 20 μg/ml PI solution containing 0.1% (v/v) Triton X-100 (Sigma) and 0.2 mg/ml DNase-free RNase A (Sigma), the cells were analyzed by FACS.

### Quantitative real time RT-PCR

Total RNA was extracted from SPDCs using TRIzol reagent (Invitrogen, Carlsbad, CA) according to the manufacturer's instructions. Complementary DNA was prepared using a reverse-transcription kit from TOYOBO (Osaka, Japan). Real-time reverse-transcription PCR (RT-PCR) was performed using a kit (SYBR Premix EX Taq, Takara) and the ABI PRISM 7300 real-time PCR system, with β-actin as an internal control. Primers used in real-time PCR were as follows: β-actin forward: CATCCGTAAAGACCTCTATGCCAAC, β-actin reverse: ATGGAGCCACCGATCCACA, HES1 forward: GCAGACATTCTGGAAATGACTGTGA, HES1 reverse: GAGTGCGCACCTCGGTGTTA; HES5 forward: AAAGACGGCCTCTGAGCAC, HES5 reverse: GGTGCTTCACAGTCATTTCCA.

### Statistics

Statistical analysis was performed with the SPSS 12.0 program. Results were expressed as means ± SD. Comparisons between groups were undertaken using the unpaired Student's *t*-test. *P *< 0.05 was considered statistically significant.

## Competing interests

The authors declare that they have no competing interests.

## Authors' contributions

FF designed and performed all the animal and cell biological experiments, carried out data analysis and drafted the manuscript. WYC assisted in cell biological experiments. HXB assisted in immunological experiments. LXW assisted in in vivo animal experiments. JG assisted in FACS analysis. CYR participated in animal breed. WL assisted in cell culture. HF assisted in cell magnetic sorting. DGR assisted in data collection. LL participated in tumor antigen preparation. ZHW assisted in experiment design. HH, as Director of the department, coordinated its execution and design, and drafted and produced the final version of the manuscript. All authors read and approved the present version of the manuscript.

## Supplementary Material

Additional file 1 **Additional Figure a1.** SPDC subpopulations in RBP-J^-/- ^and RBP-J^+/- ^mice. Click here for file

Additional file 2 **Additional Figure a2.** Infiltration of CD4^+^, CD8^+^, CD19^+ ^and NK1.1^+ ^cells in the tumors of the mice bearing the S180 and LLC tumors. Click here for file
