# Supplementary material for: The transcription factor RBP-J-mediated signaling is essential for dendritic cells to evoke efficient anti-tumor immune responses in mice
Source: Mol Cancer. 2010 Apr 27;9:90. doi: 10.1186/1476-4598-9-90 (PMC2867822; doi:10.1186/1476-4598-9-90)
Supplement: Additional file 1 — Additional Figure a1. SPDC subpopulations in RBP-J-/- and RBP-J+/- mice. [file 1476-4598-9-90-S1.DOC]

**Additional file 1**

**
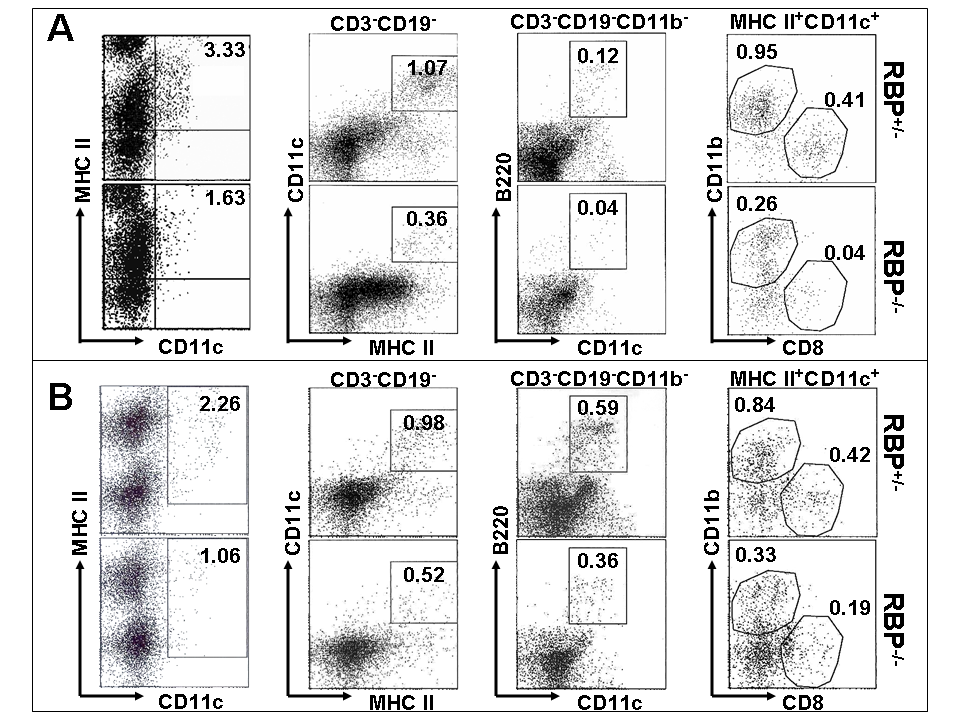
**

**Additional Figure a1. SPDC subpopulations in RBP-J^-/-^ and RBP-J^+/-^ mice.** (A) FACS analysis of spleen cells from the poly(I)-poly(C)-induced RBP-J^-/-^ and RBP-J^+/-^ mice using the indicated antibodies. (B) Bone marrow cells from RBP-J^-/-^ and RBP-J^+/-^ mice were transplanted into irradiated wild type congenic mice. One month later, the spleens were dissected from recipient mice and single-cell suspensions were prepared and analyzed by FACS as in (A).
