# Supplementary material for: The transcription factor RBP-J-mediated signaling is essential for dendritic cells to evoke efficient anti-tumor immune responses in mice
Source: Mol Cancer. 2010 Apr 27;9:90. doi: 10.1186/1476-4598-9-90 (PMC2867822; doi:10.1186/1476-4598-9-90)
Supplement: Additional file 2 — Additional Figure a2. Infiltration of CD4+, CD8+, CD19+ and NK1.1+ cells in the tumors of the mice bearing the S180 and LLC tumors. [file 1476-4598-9-90-S2.DOC]

**Additional file 2**

**
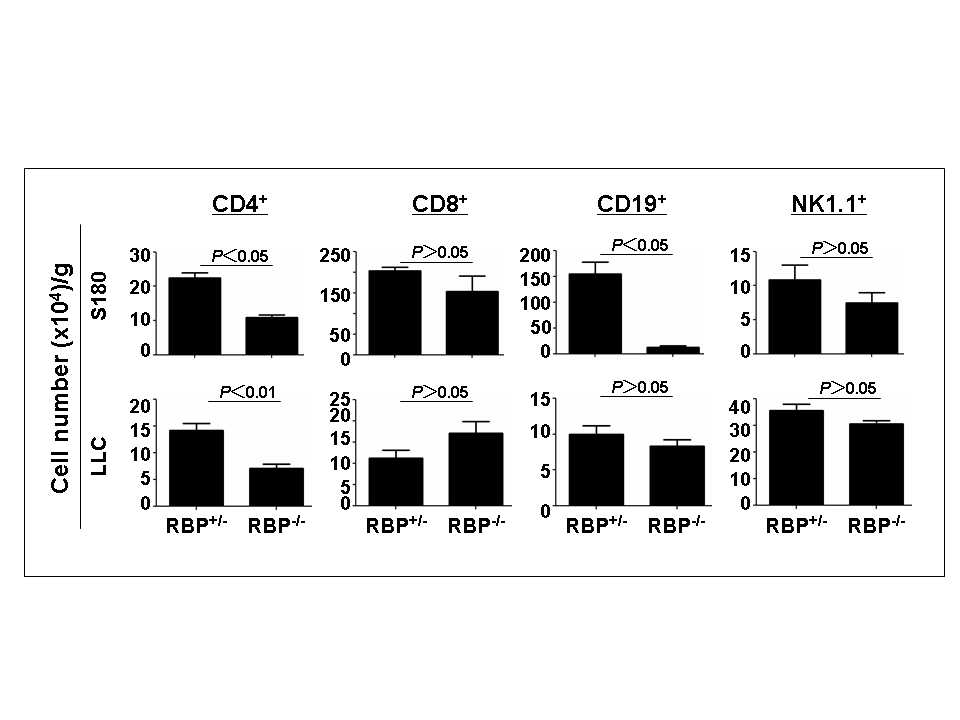
**

**Additional Figure a2. Infiltration of CD4^+^, CD8^+^, CD19^+^ and NK1.1^+^ cells in the tumors of the mice bearing S180 and LLC tumors.** The tumor cells were mixed with RBP-J^+/-^ and RBP-J^-/-^ SPDCs, and were injected subcutaneously into normal mice. The tumors were dissected on the 17th day after the inoculation, and the single-cell suspensions were prepared for FACS analyses. The number of CD4^+^, CD8^+^, CD19^+^ and NK1.1^+^ cells in 1 g of tumor tissues was calculated based on FACS.
